# Identifying the presence and severity of dementia by applying interpretable machine learning techniques on structured clinical records

**DOI:** 10.1186/s12911-022-02004-3

**Published:** 2022-10-17

**Authors:** Akhilesh Vyas, Fotis Aisopos, Maria-Esther Vidal, Peter Garrard, Georgios Paliouras

**Affiliations:** 1 L3S Research Center, Leibniz University Hannover, Hannover, Germany; 2 Scientific Data Management research group, TIB-Leibniz Information Centre for Science and Technology, Hannover, Germany; 3 Software and Knowledge Engineering Laboratory, Institute of Informatics and Telecommunications, NCSR “Demokritos”, Athens, Greece; 4 Molecular and Clinical Science Research Institute, St George’s, University of London, London, UK

**Keywords:** Dementia, Mini mental score, Machine learning, Data science, LIME, CAMCOG

## Abstract

**Background:**

Dementia develops as cognitive abilities deteriorate, and early detection is critical for effective preventive interventions. However, mainstream diagnostic tests and screening tools, such as CAMCOG and MMSE, often fail to detect dementia accurately. Various graph-based or feature-dependent prediction and progression models have been proposed. Whenever these models exploit information in the patients’ Electronic Medical Records, they represent promising options to identify the presence and severity of dementia more precisely.

**Methods:**

The methods presented in this paper aim to address two problems related to dementia: (a) Basic diagnosis: identifying the presence of dementia in individuals, and (b) Severity diagnosis: predicting the presence of dementia, as well as the severity of the disease. We formulate these two tasks as classification problems and address them using machine learning models based on random forests and decision tree, analysing structured clinical data from an elderly population cohort. We perform a hybrid data curation strategy in which a dementia expert is involved to verify that curation decisions are meaningful. We then employ the machine learning algorithms that classify individual episodes into a specific dementia class. Decision trees are also used for enhancing the explainability of decisions made by prediction models, allowing medical experts to identify the most crucial patient features and their threshold values for the classification of dementia.

**Results:**

Our experiment results prove that baseline arithmetic or cognitive tests, along with demographic features, can predict dementia and its severity with high accuracy. In specific, our prediction models have reached an average f1-score of 0.93 and 0.81 for problems (a) and (b), respectively. Moreover, the decision trees produced for the two issues empower the interpretability of the prediction models.

**Conclusions:**

This study proves that there can be an accurate estimation of the existence and severity of dementia disease by analysing various electronic medical record features and cognitive tests from the episodes of the elderly population. Moreover, a set of decision rules may comprise the building blocks for an efficient patient classification. Relevant clinical and screening test features (e.g. simple arithmetic or animal fluency tasks) represent precise predictors without calculating the scores of mainstream cognitive tests such as MMSE and CAMCOG. Such predictive model can identify not only meaningful features, but also justifications of classification. As a result, the predictive power of machine learning models over curated clinical data is proved, paving the path for a more accurate diagnosis of dementia.

**Supplementary Information:**

The online version contains supplementary material available at 10.1186/s12911-022-02004-3.

## Background

According to recent surveys, dementia is underdiagnosed. ICD codes alone cannot serve as a reliable gold standard for investigating the demographic characteristics or the clinical associations of the condition using electronic health records [1].

There is no single diagnostic test that can determine if a person has any form of dementia. However, clinicians employ various tools and tests to detect the presence of dementia, whether due to Alzheimer’s disease or some other cause. The Mini’ Mental State Examination (MMSE) is the most common test for measuring cognitive impairment [2]. Creavin et al. [3] attempt to determine the diagnostic accuracy of MMSE at various cut points for dementia in people aged 65 years and over. The authors conclude that MMSE contributes to a diagnosis of dementia in low prevalence settings. But this work also suggests that MMSE should not be used in isolation to confirm or exclude the disease. An alternative approach is to use the Clock Drawing Test (CDT) [4], which provides a simple scoring system for the rapid screening for cognitive impairment in patients with mild cognitive impairment [5].

More recently, a variety of automatic speech-based tools have also been used to detect dementia. These approaches usually employ machine learning classifiers trained with various vocal features, derived from recorded data resulting from standard spoken tasks provided to individuals [6, 7]. Automatic speech-based tools can also use lexical and conversation analysis-inspired features derived from transcripts of recorded data [8], in conversations led by neurologists or intelligent virtual agents. As another example, the CogAware tool [9] provides textual analysis for transcripts of individuals describing the “cookie theft” picture [10], in order to automatically detect whether they are originated from a patient with dementia or a cognitively normal individual.

A powerful but underutilised resource that could be employed to rapidly and automatically detect dementia is a patient’s electronic medical record (EMR). EMRs are increasingly available sources of information that contain large quantities of heterogeneous data. EMRs can include MR images of the brain that demonstrate cortical atrophy or comprise demographic and clinical information, as well as patient performance in cognitive tests. This information has been used to train machine learning models to detect the presence and severity of dementia [11–13]. Such models have also been used to assess the risk of converting to Dementia from a Mild Cognitive Impairment stage [14, 15].

Demographic and clinical information from EMRs has also been analysed by Shao et al. [1]; they utilise both structured and unstructured EMRs to define individual patients’ risk scores for dementia. The authors also combine structured data features, consisted of standard codes and medications from EMRs, with topic features, extracted from a topic modelling approach on free-text clinical notes. Finally, they employ a logistic regression model using the selected features as predictors. A similar retrospective study is combined by Ford et al. [16], using structured data including medical diagnoses, primary care tests and investigations, lifestyle information and prescribing data. Their survey compared various machine-learning models with baseline epidemiological approaches to identify undetected dementia in UK primary care patients and concludes that logistic regression and random forest algorithms allow for important features to be exposed and may be the best approaches for this prediction task.

In this paper, we examine whether the values of various features in electronic medical records can consistently decide the patient’s cognitive status, i.e. if the patient suffers from dementia and the level of its severity. This is realised in an automated way, by employing machine learning models that analyse a big dataset of EMRs, and validating the models’ effectiveness.

For this purpose, we employ EMRs that comprise structured information such as demographics, MMSE, and performance on the CAMCOG—a screening instrument for dementia. CAMCOG includes tests sensitive to different cognitive domains, and is part of the Cambridge Mental Disorders of the Elderly Examination (CAMDEX) [17]. CAMCOG has high levels of sensitivity and specificity when used to distinguish individuals with mild dementia from those who are cognitively intact. We analyse these data using a random forests model, providing an automatic classifier that effectively discriminates dementia patients from control individuals and estimates the severity of the disease for the former.

However, machine learning algorithms usually work as a black-box tool, without the ability to interpret individual predictions. Thus, an emerging challenge is to achieve the explainability of decisions taken by such models, in order to provide clinicians with the ability to understand the rationale of the model. Towards these directions, we employ decision tree models that are able to visualise a set of configurable rules for predictions made. Since MMSE and CAMCOG scores can directly provide an estimation of dementia severity, we decided to exclude those from the training of our decision trees, in order to investigate other interesting features that are clinically useful.

## Methods

### **Problem statement**

The problem addressed in this work is related to detecting the presence of a form of dementia in individuals based on a set of available demographic and psychometric features. A relevant extension of the problem above is accurately deciding the severity of the disease for patients with dementia. These two problems are formulated as follows:

1. **Problem (a)—Predict Dementia, No Dementia**: This problem addresses the issue of identifying if a patient has dementia or not in a specific episode (correctly diagnose dementia). In this task, we classify a patient-episode into “Dementia (1)” or “No-Dementia (0)”. We also identify relevant features for the classification decision.
2. **Problem (b)—Predict No Dementia, Minimal or Mild Dementia, and Moderate or Severe Dementia**: We further refine the previous problem to have a better understanding of the severity of dementia. We classify dementia patients into two more classes, “Minimal or Mild” and “Moderate or Severe”. We discover the important features behind the classification decision.

### **Approach**

Our approach initially demands a data cleaning and curation process. Since all real-world clinical datasets contain a big amount of noise and missing values, we need to define a set of general rules, in order to be able to complete missing features and ignore features that are sparse. This process includes the filling of stable parameters throughout the patient’s lifetime, such as height, and removing parameters where the majority of values in patient episodes are meaningless (e.g. ‘not known’ or ‘not asked’).

The problems previously defined are formulated as simple classification tasks, addressed by machine learning models. These models’ algorithms are based on a supervised learning Decision Tree (DT) method to make the decisions easily interpretable by clinicians. They predict the class of patients by inferring decision rules from training data features.

A decision tree is composed of nodes and leaves. A node represents a dichotomous threshold for the value of some feature in the dataset (a.k.a. decision threshold). A leaf represents a patient subgroup in whom the likelihood of belonging to the positive class (in this case, developing dementia) cannot be refined by any additional dichotomous test. Nodes and leaves are connected by branches, each of which represents an additional condition; any path through the decision tree represents the outcome of a series of conditional statements.

**Fig. 1:**
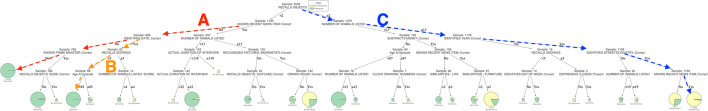
The decision tree predicts the dementia classes (“Dementia”/“No Dementia”) of 2505 patients for the Problem (a). The pie charts in leaves show class labels, the proportion of resulting classes and their support size. The branches demonstrate connections between features and their threshold values, leading towards class labels. In the tree, some most significant paths A, B and C predict nearly pure decisive classes for large patients. The rules of path A and path B lead to a pure “Dementia” population of 648 and 57 patients, respectively, while the rules of path C lead to a large majority of “No Dementia” population comprised by 1092 individuals. (see Additional file 1: Figure S1 in Supplementary Information for the better visibility)

To better understand the concept of decision trees, consider the example tree in Fig. 1. In this tree, the goal is to decide whether an individual has some form of dementia or not (binary classification). As we can see, each intermediate node entails a discriminating feature and a threshold. Based on the value of this feature, a clinician must follow the appropriate branch, until reaching a leaf node. Leaf nodes conclude to a decision (“Dementia” / “No Dementia”), based on the majority of cases in the training sample. To make our trees more informative and self-explainable, we have visualized the percentages in pie charts, the size of which depends on the number of cases falling in this leaf node. It is also used to find the class probability, which is the fraction of the same class in a leaf.

#### **Predictive models**

Our objective is to predict the classification of dementia patients as described in the problem statement, based on various patient record parameters provided as features in machine learning-based predictive models. We utilize the random forest algorithm for class prediction and the local interpretable model-agnostic explanations (LIME) [18] for explaining the model decision for any patient-episode. As a last step, we train a decision tree classifier, feeding important data features obtained by the random forest classifier model.

A random forest algorithm is an ensemble learning method that generates different decision trees. The decision of an algorithm averages the results provided by different decision trees. Individual decision trees usually have high variance and are prone to overfitting. Therefore, to control overfitting, decision trees are trained on different sub-samples of the dataset instances. A random forest model does not provide an explanation for each individual test instance classification. However, it gives a list of global important features based on the complete sample of the training data considering impurity. To determine the importance of a feature, the random forest model measures how much this feature impacts the total reduction of the classification criterion, i.e. how discriminating this feature is for the data instances to candidate classes. The greater the number, the more important the feature.

**Fig. 2:**
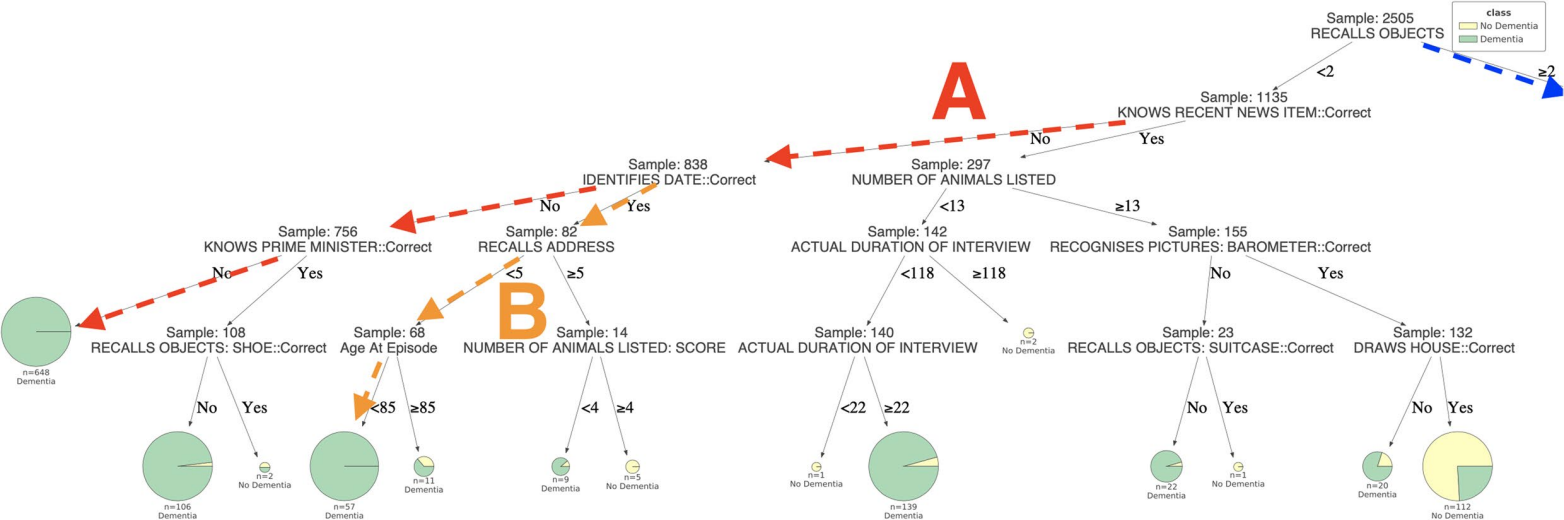
The left part of the decision tree (Fig. 1) predicts the dementia classes (“Dementia”/“No Dementia”) of 2505 patients for the Problem (a). In the tree, some most significant paths A and B predict nearly pure decisive classes for large patients. The rules of path A and path B lead to a pure “Dementia” population of 648 and 57 patients, respectively

**Fig. 3:**
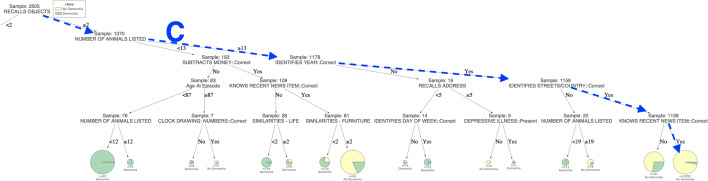
The right part of the decision tree (Fig. 1) predicts the dementia classes (“Dementia”/“No Dementia”) of 2505 patients for the Problem (a). In the tree, one of the most significant paths C predicts nearly pure decisive classes for large patients. The rules of path C lead to a large majority of “No Dementia” population, comprised by 1092 individuals

In contrary, LIME provides a local explanation of the prediction for each instance of the complete sample of the test data. LIME utilizes local surrogate models to explain the black-box behaviour of the machine learning model and its prediction. In terms of weight, LIME calculates each feature contribution for the predicted class of a test instance. As a result, the contribution or weight of each feature may vary depending on the test instance (local explainability of the prediction). The relevance of the feature is reflected on its weight. The weight’s importance can be interpreted by applying this to the prediction probability of a predicted class (Figs. 2, 3).

### **Experiments**

#### **Dataset**

In our experiments, we employ clinical data from the OPTIMA (Oxford Project to Investigate Memory and Ageing) [19–21] dataset. The OPTIMA project was a long-term cohort study (1988–2008) of ageing and dementia that included persons over 70 with normal or minimally impaired cognition and studied their physical, metabolic, imaging, clinical, and cognitive indices until death.

The OPTIMA dataset comprises 1035 different patients with 9584 episodes and their features documented. The collection also includes unique patient identifiers; they, when combined with episode dates, uniquely identify each assessment of a patient’s status. Each episode has 1593 distinct features. The features are derived from various sources of information, including demographic characteristics, YES/NO questions related to health and well-being, rating scales, medical history, physical examinations, neuropsychological assessments, and performance of cognitive tests. As will be concluded, only a subset of these features will be important for the prediction tasks.

An important issue in OPTIMA dataset, is that 62% of the values are missing. This occurs due to a variety of reasons. For example, clinicians may note down only the most relevant feature values about a patient’s current condition and neglect others. They may also ignore some repeated feature across episodes, such as demographics and prolonging comorbidities (diabetes or hypertension). Based on this, we devise pre-processing and cleaning techniques able to curate missing values based on the supervision of our clinicians.

#### **Determining classes for the predictive model**

The predictive models are trained on patient-episode data (a.k.a. data instance). We note that there are substantial differences between each patient’s different episode features. In specific, our models use two kinds of features that change between episodes as follows:

- Demographic (e.g. age or weight) or clinical features (e.g. comorbidities). The majority of these values usually change between two episodes (especially for long time intervals).
- Cognitive examinations or memory and arithmetic tests (e.g. RECALLS OBJECTS, SUBTRACTS MONEY), taken by patients in each episode with the supervision of a clinician. Resulting values can be much different even in consecutive episodes, as the cognitive state of the individual deteriorates.

As a result, we avoid the potential information leakage introduced when the same episode or patient episodes with the same values could be part of both training and test data.

There are no direct features to suggest if a patient episode is labelled as “Dementia” or “No Dementia”. Therefore, supported by our clinicians, we define our ground truth, and label each data instance based on the features with values illustrated in Tables 1, 2, and 3. Features and values in Tables 1, 2 are used to identify if a patient episode falls under the “Dementia” or “No Dementia” classes, respectively. In addition, 3385 episodes are dropped because all their values are NULL, None or Unknown in the class determining features ( in Tables 1, 2, and 3). Moreover, a patient-episode is labelled as “Dementia”, if and only if, any other episode of the same patient is labelled as dementia, according to the criteria of the ground truth. Therefore, all the patient’s episodes fall into one of two categories: “Dementia” or “No Dementia”. Features and values in Table 3 are used to label a data instance with the value of the dementia severity (Minimal, Mild, Moderate, Severe). In case of several labels, the one representing the higher severity is considered.

**Table 1:** Features and their respective values to consider a patient in dementia class

Features considered	Values considered	Total cases
EST SEVERITY OF DEMENTIA	Minimal, mild, moderate, severe	2552
DEMENTIA CLOUDED	Present	10
CLOUDED DEMENTIA	Present	2
SEVERITY OF DEMENTIA	Minimal, mild, moderate, severe	814
DEMENTIA PRESENT	Mild, moderate, severe	951
MIXED DEMENTIA	Yes	172
DSM-IIIR	Dementia	1
CLINICAL DIAGNOSIS 1	Dementia	876

**Table 2:** Features and their respective values to consider a patient in no-dementia class

Features considered	Values considered	Total cases
EST SEVERITY OF DEMENTIA	No	2084
SEVERITY OF DEMENTIA	No	958
DEMENTIA PRESENT	No	2185
DSM-IIIR	No dementia	1
CLINICAL DIAGNOSIS 1	No dementia	965

**Table 3:** Features and their respective values to consider the severity of dementia

Features considered	Values considered	Total cases
EST SEVERITY OF DEMENTIA	Minimal, mild, moderate, severe	2552
SEVERITY OF DEMENTIA	Minimal, mild, moderate, severe	814

#### **Data cleaning and pre-processing**

Various related works examined in [22] show that features related to demographics (e.g. age, gender and education), health (e.g. BMI, diabetes, depression, high cholesterol, and traumatic brain injury), and lifestyle factors (e.g. smoking, alcohol, physical activity, cognitive activity, and fish intake) are essential in the diagnosis of dementia. We identify 242 such features in the OPTIMA dataset. Applying the curation rules suggested by our clinicians, we are able to complete a few of the missing values on some features. This can be done by adding values implicitly provided by other closely related features. Curated features include “Petersen MCI”, “Depressive Illness”, “Cerebro Vascular Disease Present”, and “Anxiety/Phobic”. For example, our clinical experts can first identify closely related features for the “Depressive Illness” feature (with possible values: “absent” and “present”); these features include “Severity of depression”, “Feeling depressed”, “Depressed mood” and “Depression/Dysphoria: Severity”). The missing values of the ‘Depressive Illness” feature, are filled by taking into account values of the related features. For example, “Mild” or “Moderate” from “Depression/Dysphoria: Severity” entails the value “present” in “Depressive Illness”. Using these curation rules, around 70% of the missing values are completed.

The OPTIMA dataset feature-guide is included in the Additional file 5: Table S1 in supplementary information section. It is used to identify meaningless values in the data features. It consists of feature labels, their descriptions (data types and format of features), including the range of numerical features and categories of categorical features. We replace all such meaningless values with missing values (NULL). For example, “IDENTIFIES YEAR” feature should have ‘YES’ and ‘NO’ values only, so all other cases are considered as missing values.

As data sparsity can cause improper learning to the models, we decided to drop some dataset episodes and features, according to the number of missing values. An episode is removed if at least 50% of the feature values are missing. On the other hand, a full feature is removed if at least 5% of the episodes have missing values in this feature. Those percentages were chosen after experimentation with different thresholds, as they exhibited the best performance possible. This whole procedure leaves our models with the features mentioned in Table 4 for Problem (a) and Problem (b). Further, during pre-processing, categorical features are transformed into one-hot encoding. After the curation process, we end up with 3579 data instances (episodes) to perform experiments for Problem (a) and Problem (b).

**Table 4:** Selected features for Problem (a) and (b)

RECALLS OBJECTS	CLOCK DRAWING	SUBTRACTING SEVENS
SIMILARITIES - FRUIT	NUMBER OF ANIMALS LISTED: SCORE	NUMBER OF ANIMALS LISTED
SIMILARITIES - LIFE	IDENTIFIES OBJECTS	MIME - BRUSHING TEETH
RECALLS ADDRESS	dementia range	PRAXIS - PAPER
RECOGNISES OBJECTS	IDENTIFIES COIN	SIMILARITIES - CLOTHING
PATIENT	RECALLS OBJECTS	REGISTERS OBJECTS
RECOGNISES FAMOUS PEOPLE	SIMILARITIES - FURNITURE	ACTUAL DURATION OF INTERVIEW
Age At Episode	RECALLS ADDRESS: BROWN	PRAXIS - ENVELOPE
IDENTIFIES FLOOR	KNOWS PRIME MINISTER	COMPREHENDS TAP
RECALLS OBJECTS: BAROMETER	RECOGNISES OBJECTS: PIPE	REPETITION
REMEMBERS WW2 DATE	IDENTIFIES MONTH	DRAWS HOUSE
CLOCK DRAWING: TIME	RECOGNISES PICTURES: SCALES	RECALLS OBJECTS: SHOE
RECALLS ADDRESS: JOHN	DEFINES HAMMER	RECOGNISES PICTURES: SHOE
IDENTIFIES OBJECTS: PENCIL	PRAXIS - PAPER: FOLDS	WRITES A SENTENCE
READING COMPREHENSION 2	REGISTERS OBJECTS 1: APPLE	DICTATION
DICTATION::Poor	COMPREHENDS RADIO	RECOGNISES PICTURES: BAROMETER
IDENTIFIES DATE	IDENTIFIES YEAR	IDENTIFIES STREETS COUNTRY
DRAWS PENTAGON	COMPREHENDS VILLAGE	RECALLS OBJECTS: TYPEWRITER
RECOGNISE PERSON	IDENTIFIES COUNTY	REMEMBERS MAE WEST
KNOWS MONARCH	RECALLS ADDRESS: D42	RECALLS OBJECTS: SUITCASE
COUNTING BACKWARDS: > two errors	COUNTING BACKWARDS	COUNTING BACKWARDS::One error
KNOWS RECENT NEWS ITEM	DEPRESSIVE ILLNESS::Absent	DEPRESSIVE ILLNESS::Present
RECOGNISES OBJECTS: SHOE	COMPREHENDS NOD	RECOGNISES OBJECTS: TELEPHONE
REGISTERS OBJECTS 3: PENNY	MIME - SCISSORS	MIME - SCISSORS::Poor
RECALLS ADDRESS: WEST	KNOWS HEIR TO THRONE	NAMES PICTURES: LAMP
RECOGNISES OBJECTS: PURSE	CLOCK DRAWING: NUMBERS	RECALLS OBJECTS 3: PENNY
CLOCK DRAWING: CIRCLE	PRAXIS - PAPER: RIGHT HAND	READING COMPREHENSION 1
REMEMBERS HITLER	PRAXIS - PAPER: ON LAP	DIAGNOSIS 334-351: ANXIETY PHOBIC::Absent
DIAGNOSIS 334-351: ANXIETY/PHOBIC::Present	RECALLS OBJECTS: LAMP	REMEMBERS LINDBERGH
NAMES PICTURES: TYPEWRITER	RECOGNISES OBJECTS: CUP	IDENTIFIES TOWN
IDENTIFIES OBJECTS: WATCH	COMPREHENDS TOUCH	COMPREHENDS HOTEL
NAMES PICTURES: SHOE	DRAWS SPIRAL	RECALLS ADDRESS: BEDFORD
SUBTRACTS MONEY	RECALLS OBJECTS 2: TABLE	NAMES PICTURES: BAROMETER
IDENTIFIES SEASON	MIME WAVE	RECOGNISES OBJECTS: SPECTACLES
REMEMBERS STALIN	IDENTIFIES PRESENT PLACE	ADDS UP MONEY
COMPREHENDS LOOK	NAMES PICTURES: SCALES	NAMES PICTURES: SUITCASE
REMEMBERS WW1 DATE	RECALLS OBJECTS 1: APPLE	IDENTIFIES DAY OF WEEK
RECALLS OBJECTS: SCALES		

### **Model training and feature selection**

We employ a random forest classifier to train our predictive models with the default parameters of sklearn library,^1^ except for the maximum depth of the tree, which is set to 5. A random forest model utilises a stratified shuffle split cross-validator which splits data into 5 folds of train and test set with reshuffling. Each fold preserves the percentage of samples for each class. After each fold, we record 50 most impurity-based important features from the model training and store into a set to have a distinct feature list. These important features are utilised to train a decision tree classification model to enhance interpretability of the random forest model outcomes. A maximum depth of 5 is used to generate generalised decision trees without overfitting.

Following a most common approach as per the empirical study, we choose the split ratio (70:30) between the training and testing set and utilise a stratified method to preserve class frequencies in both sets. Both problems represent the same experimental settings. In Problem (a), the “No Dementia” and “Dementia” classes contain 1829 and 1750 episodes, respectively, while, in Problem (b), the “No Dementia”, “Minimal or Mild dementia”, and “Moderate or Severe dementia” classes have 1829, 1281, and 469 episodes, respectively. The population size of dataset (Train:Test) in both problems are equal to 3579 (2505:1074) patient episodes.

Tables 5, 6, 7, 8, 9 and Table 10 report the results of Problem (a) and Problem (b), respectively, using the random forest and decision tree algorithms.

**Table 5:** Evaluation results for Problem (a) (Predict No-Dementia and Dementia) with 5-fold cross validation after each iteration of a random forest model in terms of macro-averaged precision, recall, and f1-score, where training and testing set are divided based on patient-episode setting

Iterations No.	Precision	Recall	f1-score
1	0.95	0.95	0.95
2	0.96	0.96	0.96
3	0.94	0.94	0.94
4	0.96	0.97	0.96
5	0.95	0.95	0.95

**Table 6:** Evaluation results for Problem (a) (Predict No-Dementia and Dementia) after each different random iteration of a random forest model in terms of macro-averaged precision, recall, and f1-score, in the patient-level setting

Iterations No.	Precision	Recall	f1-score
1	0.96	0.96	0.96
2	0.95	0.95	0.95
3	0.95	0.95	0.95
4	0.96	0.95	0.95
5	0.96	0.96	0.96

**Table 7:** Evaluation results for Problem (b) (Predict No Dementia, Minimal or Mild Dementia and Moderate or Severe Dementia) with 5-fold cross validation after each iteration of a random forest model in terms of macro-averaged precision, recall and f1-score, where training and testing set are divided based on patient-episode setting

Iterations No.	Precision	Recall	f1-score
1	0.88	0.85	0.86
2	0.86	0.81	0.83
3	0.88	0.84	0.85
4	0.88	0.84	0.85
5	0.89	0.85	0.86

**Table 8:** Evaluation results for Problem (b) (Predict No Dementia, Minimal or Mild Dementia and Moderate or Severe Dementia) after each different random iteration of a random forest model in terms of macro-averaged precision, recall and f1-score, in the patient-level setting

Iterations No.	Precision	Recall	f1-score
1	0.83	0.78	0.80
2	0.85	0.80	0.82
3	0.86	0.81	0.83
4	0.86	0.84	0.85
5	0.87	0.81	0.83

**Table 9:** Classification report: evaluation results for Problem (a) (Predict No-Dementia and Dementia) using a decision-tree model in terms of precision, recall and f1-score

	Decision tree model			
	Precision	Recall	f1-score	Support
No-Dementia	0.91	0.97	0.93	549
Dementia	0.96	0.90	0.93	525
macro avg	0.93	0.93	0.93	1074
weighted avg	0.93	0.93	0.93	1074

The support represents the number of true instances of each class. The macro average and weighted average calculate the metrics for each class label. However, the macro illustrates the unweighted mean, without considering label imbalance, whereas the weighted average utilises the support of labels for producing the weighted mean value

**Table 10:** Classification Report: Evaluation results for Problem (b) (Predict No Dementia, Minimal or Mild Dementia and Moderate or Severe Dementia) using a decision-tree model, in terms of precision, recall and f1-score

	Decision tree model			
	Precision	Recall	f1-score	Support
No-Dementia	0.91	0.97	0.94	549
Minimal or Mild Dementia	0.81	0.78	0.79	384
Moderate or Severe Dementia	0.74	0.65	0.69	141
macro avg	0.82	0.80	0.81	1074
weighted avg	0.85	0.86	0.85	1074

The support represents the number of true instances of each class. The macro average and weighted average calculate the metrics for each class label. However, the macro illustrates the unweighted mean, without considering label imbalance, whereas the weighted average utilises the support of labels for producing the weighted mean value

*Evaluation Metrics* We measure the performance of the predictive models in terms of precision, recall, and f1-score. In Problem (a), precision is the ratio of correctly predicted patients in the “Dementia” class to the total patients predicated as dementia. Alternatively, recall represents the ratio between the number of patients correctly predicted in the “Dementia” class to all patients in the “Dementia” class. Lastly, f1-score is the harmonic mean of precision and recall. The same metrics are also utilised for the classification of patients into the “No Dementia” class. We do not take accuracy (ratio between total correctly predicted patients to the total patients) into the account, as it is the least significant compared to the f1-score. We use the same metrics for Problem (b).

## Results

The random forest predictive model’s performance is measured after each fold in terms of macro-averaged precision, recall, and f1-score of each class. We represent the results of the random forest models in Tables 5 and 7 for Problem (a) and Problem (b) considering patient episodes as data instances into the training and testing set.

In order to validate that the considering several episodes of the same patient does not induce overfitting, the models are also trained and validated at patient-level. In the patient-level setting, each patient record that includes all his/her episodes is included either in the training or testing sets but, not in both.

Tables 6 and 8 report on the results for Problem (a) and Problem (b) respectively for patient-level setting. The performance of the predictive models is very similar at patient-episode and patient-level setting. This suggests that treating each patient’s episode as a data instance does not introduce overfitting into the models.

Moreover, we also show the results of the decision tree models for the two problems in Tables 9 and 10, respectively. The decision tree predictive model for Problem (a) provides similar results for both classes. The decision tree predictive model for Problem (b) accomplishes better results for “No Dementia” and “Minimal or Mild dementia” classes, compared to “Moderate or Severe dementia” classes. The outcomes reported in Tables 9 and 10 suggest that Problem (b) is more complex to solve than Problem (a). The complexity is introduced because the “Dementia” class is divided into the classes “Minimal or Mild” and “Moderate or Severe” dementia classes for Problem (b), which causes a high imbalance between the three classes.

*Baseline Results* We built our own baseline to compare the findings of our model. Our proposed methodology comprises the following cleaning and preprocessing steps:

1. Imputing missing values, computed from meaningless values and based on curation rules provided by the clinicians.
2. Selection of relevant features, guided by clinicians’ recommendations.
3. Identifying and replacing corrupted and meaningless values in features with null values, as well as removing problematic features.
4. One-hot encoding of categorical features for better explainability for the models

We define our baseline as a plain classification model that does not include these steps. The dataset for this model consists of 4536 episodes for training (70%) and testing(30%) for Problem (a) and Problem(b) using decision tree model. Results of the baseline model for the two problems are presented in Tables 11 and 12, respectively. We find no significant differences in precision, recall, or f1-score. However, there are differences in the decision trees generated by our baseline and our proposed model. In the supplementary information section, we provide the baseline decision trees in files (Additional file 3: Figure S3 and Additional file 4: Figure S4). The decision trees differ both in appearing features and in decision thresholds of certain features that cannot be interpretable. For Problem (a), out of nineteen features, the following three have non-interpretable decision thresholds:

- HANDED: Decision threshold=5 (in two nodes)
- WRITES A SENTENCE: Decision threshold=5

As can be observed in the OPTIMA feature guide, the “HANDED” feature is categorical with values 1, 2, 3, 8, 9, reflecting the handwriting capability of the person. Values 8 and 9 represent ’NotKnown’ and ’NotAsked’ respectively; according to our curation rules, they are considered meaningless. On the other hand, the “WRITES A SENTENCE” feature is binary. In none of these cases, the decision threshold value is interpretable, with respect to the meaning of the feature.

**Table 11:** Classification report: evaluation results for Problem (a) (Predict No-Dementia and Dementia) using decision-tree model in terms of precision, recall and f1-score for baseline

	Decision tree model			
	Precision	Recall	f1-score	Support
No-Dementia	0.90	0.94	0.92	677
Dementia	0.94	0.89	0.92	684
macro avg	0.92	0.92	0.92	1361
weighted avg	0.92	0.92	0.92	1361

**Table 12:** Classification report: evaluation results for Problem (b) (Predict No Dementia, Minimal or Mild Dementia and Moderate or Severe Dementia) using decision-tree model in terms of precision, recall and f1-score for baseline.

	Decision tree model			
	Precision	Recall	f1-score	Support
No-Dementia	0.92	0.95	0.94	677
Minimal or mild dementia	0.78	0.73	0.76	423
Moderate or severe dementia	0.73	0.74	0.74	261
Macro avg	0.81	0.81	0.81	1361
Weighted avg	0.84	0.84	0.84	1361

Similarly, in the baseline model decision tree for Problem (b), out of twenty-five features, the following four binary ones have non-interpretable decision thresholds:

- KNOWS RECENT NEWS ITEM: Decision threshold=5
- RECALLS OBJECTS 1 APPLE: Decision threshold=6
- COGNITIVE IMPAIRMENT: Decision threshold=5
- CERBRO-VASCULAR DISEASE PRESENT: Decision threshold=5

The aforementioned unmeaningful decision boundaries for both problems, appear as a result of the large amount of noise and missing values in the OPTIMA dataset for many important features. Contrary, in the decision trees of our model where data have been curated according to the preprocessing steps 1–4, none of the decision thresholds suffers from this problem. In all cases, the decision thresholds are meaningful, since all decisions taken are based on the different categories of each feature range of values.

### **Interpretation of results**

**Fig. 4:**
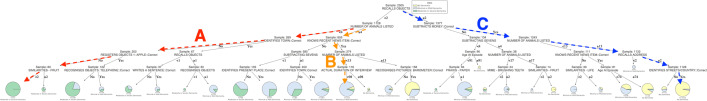
The decision tree predicts the dementia classes (“No Dementia”/ “Minimal or Mild Dementia” / “Moderate or Severe Dementia”) of 2505 patients for the Problem (b). The pie charts in leaves show class labels, the proportion of resulting classes and their support size. The branches demonstrate connections between features and their threshold values, leading towards class labels. In the tree, some most significant paths A, B and C predict nearly pure decisive classes for patients. The rules of path A and lead to a pure “Moderate or Severe Dementia” population of 71 patients. The rules of paths B and C lead to a large majority of “Mild or Minimal Dementia” and “No Dementia” populations, comprised by 113 and 1111 individuals, respectively (see Additional file 2: Figure S2 in Supplementary Information for the better visibility)

The resulting Decision Trees for Problems (a) and (b) are provided in Figs. 1 and 4, respectively.

The decision tree of Fig. 1, first separates the group into two main groups based on the strength of their verbal recall. Group one (n = 1370) (Fig. 3) recalled two or more of the six items (e.g. a shoe, a typewriter, a set of scales, a suitcase, a barometer, and a lamp), pictures of which they were asked to name and remember at the beginning of the CAMCOG.

Group two (n = 1135) (Fig. 2) recalled one or zero of these items. Not surprisingly, a large majority (around 90%) of the latter group are judged to suffer from dementia, while in the former the dementia diagnosis rate is only 16%. These distributions can be refined slightly by comparing the outcomes in groups based on other aspects of the assessment. For instance, the probability of dementia in a member of group two is much lower (40%), if the patient is aware of any recent news item and generates thirteen or more items in the ‘animal fluency’ task. Such patients would have had an isolated memory impairment that did not impair their independence, and would, therefore, probably have met the criteria for amnestic mild cognitive impairment (MCI). However, patients of group two may fall into a pure Dementia class by following the specific rules defined in paths A or B, illustrated in the decision tree 1. Patients in path A were not oriented to time and were not aware of any recent news item, while also not recalling basic recent public figures like the prime minister. Patients in path B were also not aware of any recent news item, but were oriented to time. However, although being less than 85 at the time of the episode, they seem unable to recall very recent information (in specific, all five elements of an address that they were asked to put on an envelope a few minutes ago).

Similarly, those in group one, who generated less than 13 items on animal fluency and failed a mental arithmetic task, had a 95% chance of suffering from dementia. Contrary, the dementia risk was around 45% in those who generated less than 13 animals, but passed mental arithmetic. The patients in group one, who had the lowest risk (5%) of dementia, were those who generated 13 or more animals and were basically oriented to time and place (identifying the current year, their country and streets). They were also aware of recent news items, as can be seen following path C.

**Fig. 5:**
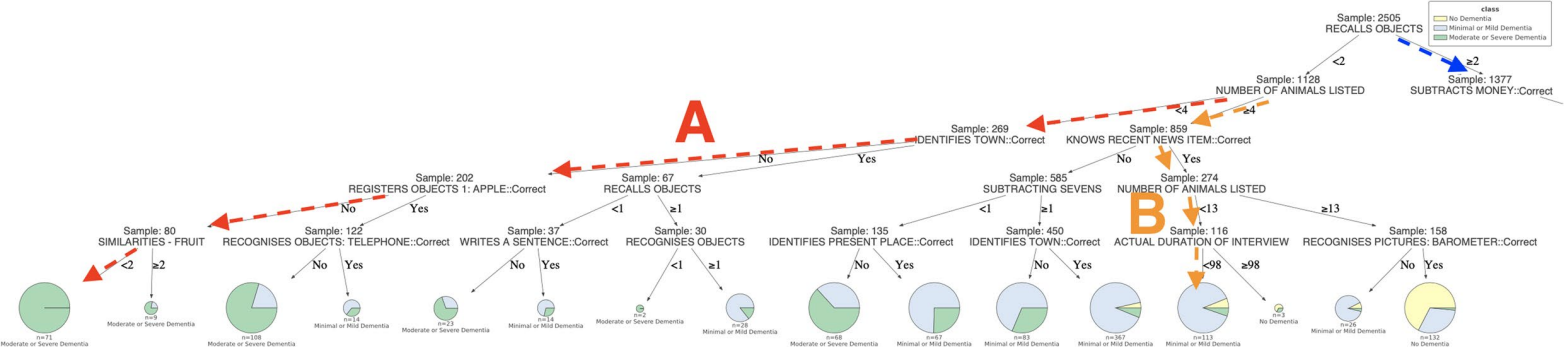
The left part of the decision tree (Fig. 4) predicts the dementia classes (“No Dementia”/ “Minimal or Mild Dementia” / “Moderate or Severe Dementia”) of 2505 patients for the Problem (b). In the tree, some most significant paths A and B predict nearly pure decisive classes for patients. The rules of path A lead to a pure “Moderate or Severe Dementia” population of 71 patients. While the rules of paths B leads to a large majority of “Mild or Minimal Dementia” populations, comprised by 113 individuals, respectively

In the decision tree of Fig. 4, the outcome of the classification is changed, such that, diagnoses of dementia are divided into those with minimal or mild dementia, and those with moderate to severe manifestations of the condition. As can be observed, the large majority of the moderate to severe cases are among the 1128 participants on the left tree branch (Fig. 5), scoring less than two on the verbal recall feature (A sub-test of the CAMCOG). However, the risk of severe dementia in these individuals ranges from 5% in those who list four or more animals and are aware of any recent news item, to 87% when three or fewer animals are generated and the town that they are in cannot be recalled.

For patients of the former group, path B rules lead to the highest possibility of minimal or mild dementia. For patients of the latter group, an absolute probability of moderate or severe dementia is provided if they are not able to recognise simple object such as an apple and cannot identify similarities for at least two kinds of fruits, as shown in path A.

**Fig. 6:**
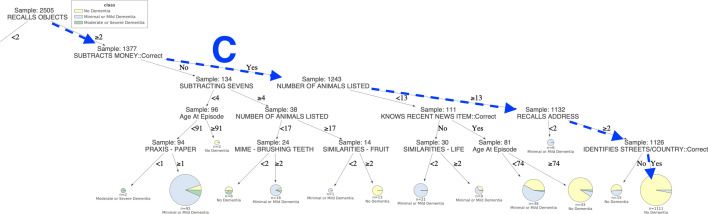
The right part of the decision tree (Fig. 4) predicts the dementia classes (“No Dementia”/ “Minimal or Mild Dementia” / “Moderate or Severe Dementia”) of 2505 patients for the Problem (b). In the tree, one of the most significant paths C predicts nearly pure decisive classes for patients. The rules of C leads to a large majority of “No Dementia” populations, comprised by 1111 individuals, respectively

Likewise, almost all cases of severe dementia in the group with better verbal recall (two or more items) occur in patients who fail a basic mental arithmetic task. Conversely, the group (Fig. 6) who are at least risk (less than 5%) of dementia of any degree are those with both accurate mental arithmetic and good verbal fluency (13 or more animals generated), as well as orientation (able to recall their address and identify their country and basic streets), as can be seen following path C.

**Fig. 7:**
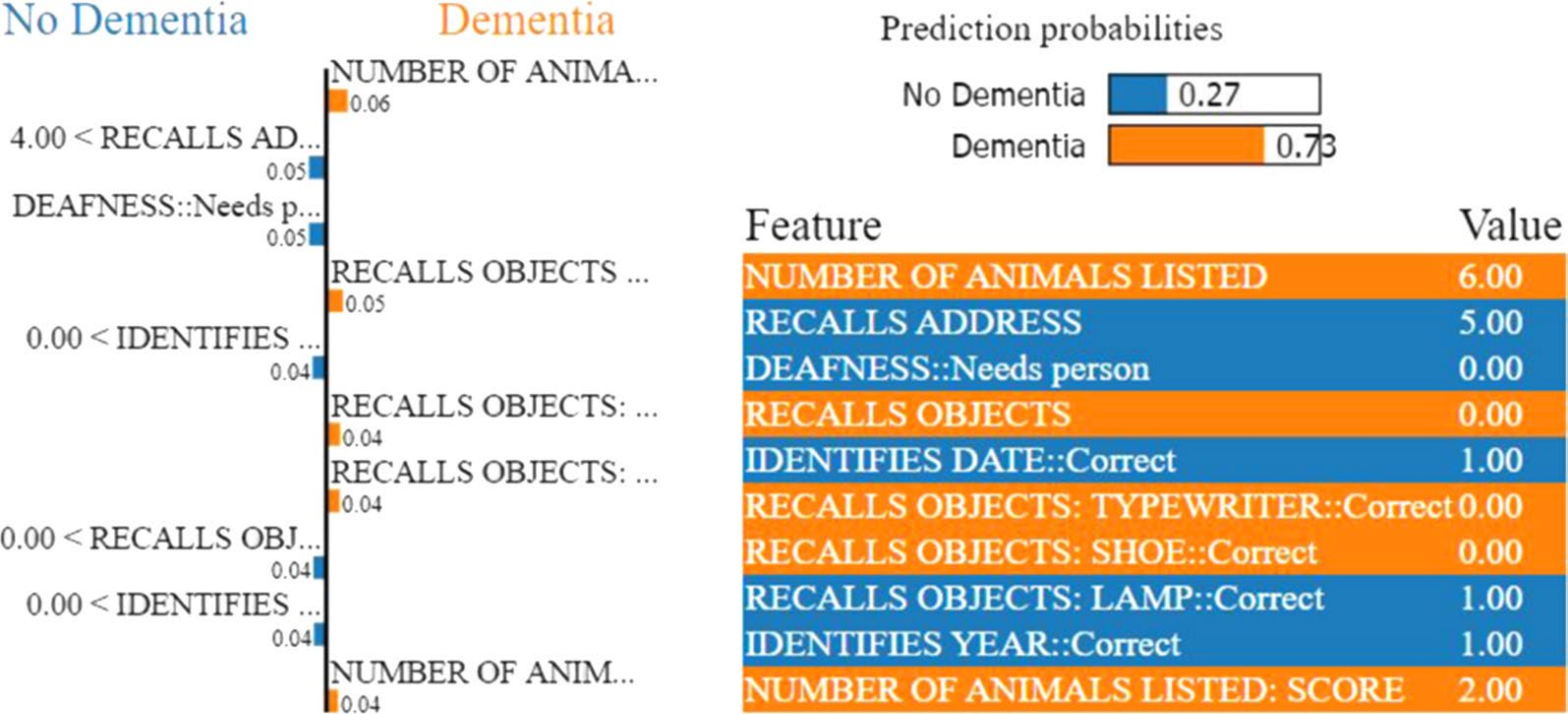
LIME provides the local interpretability for the prediction of an arbitrary test instance with prediction probabilities for the Problem (a). The left part of the figure shows the weights of the top 10 important features while making class decision, whereas the right part represents predicted class probability and the top 10 important feature with their values. The weight of the feature represents its importance. Here, the short names of the impacting features in the left part of the figure are in the same order as in the feature & value list in the right part

### **Model explanation**

To understand the machine learning model’s black-box behaviour, we perform local model interpretation over our random forest model using LIME (Local Interpretable Model-Agnostic Explanations) [18]. We also make use of a decision tree classifier model for explaining the reasons behind our predictions. Here, we consider two examples from each problem’s testing set to understand the decisions of our random forest model.

**Fig. 8:**
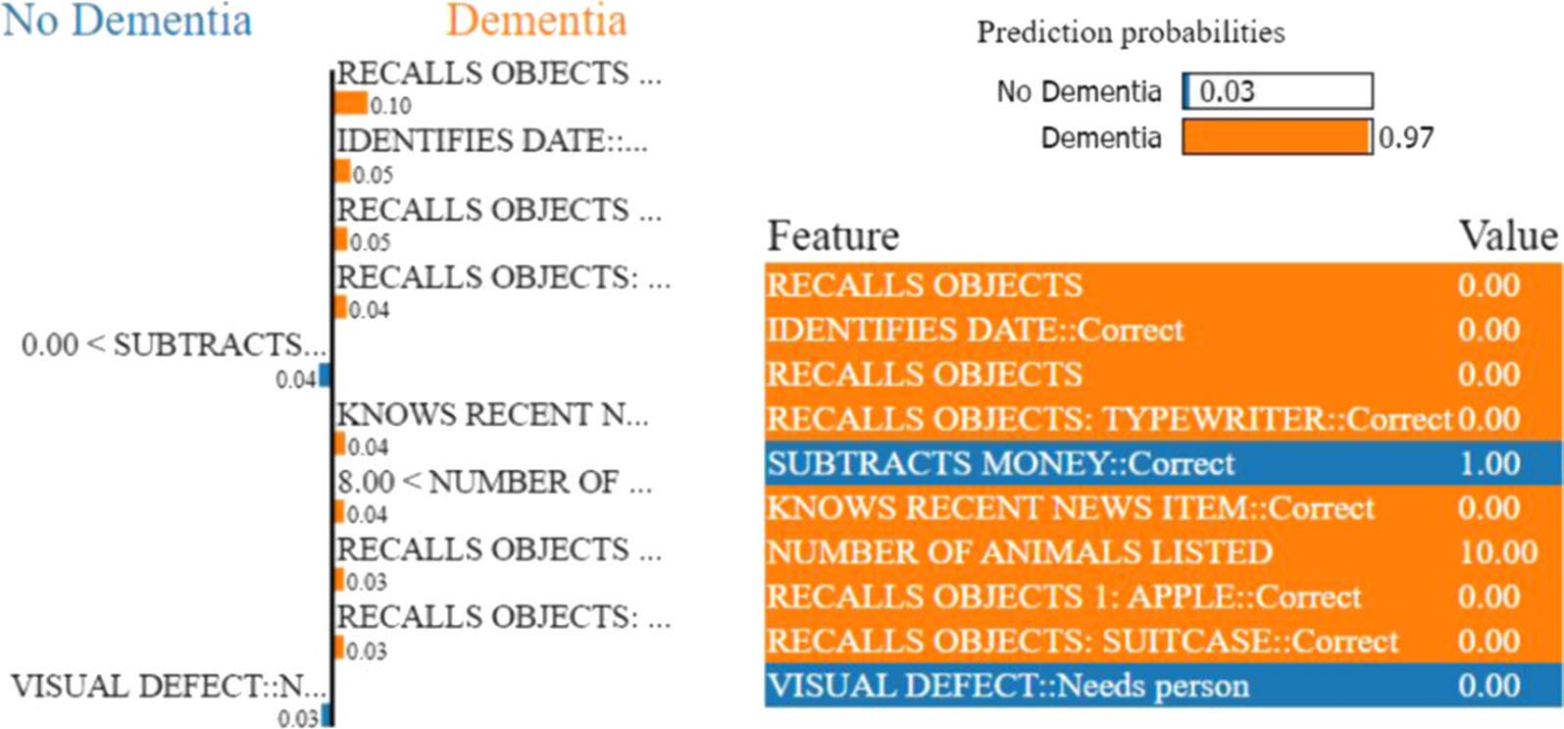
LIME provides the local interpretability for the prediction of an arbitrary test instance with prediction probabilities for Problem (a). The left part of the figure shows the weights of the top 10 important features while making class decision, whereas the right part represents predicted class probability and the top 10 important feature with their values. The weight of the feature represents its importance. Here, the short names of the impacting features in the left part of the figure are in the same order as in the feature & value list in the right part

**Fig. 9:**
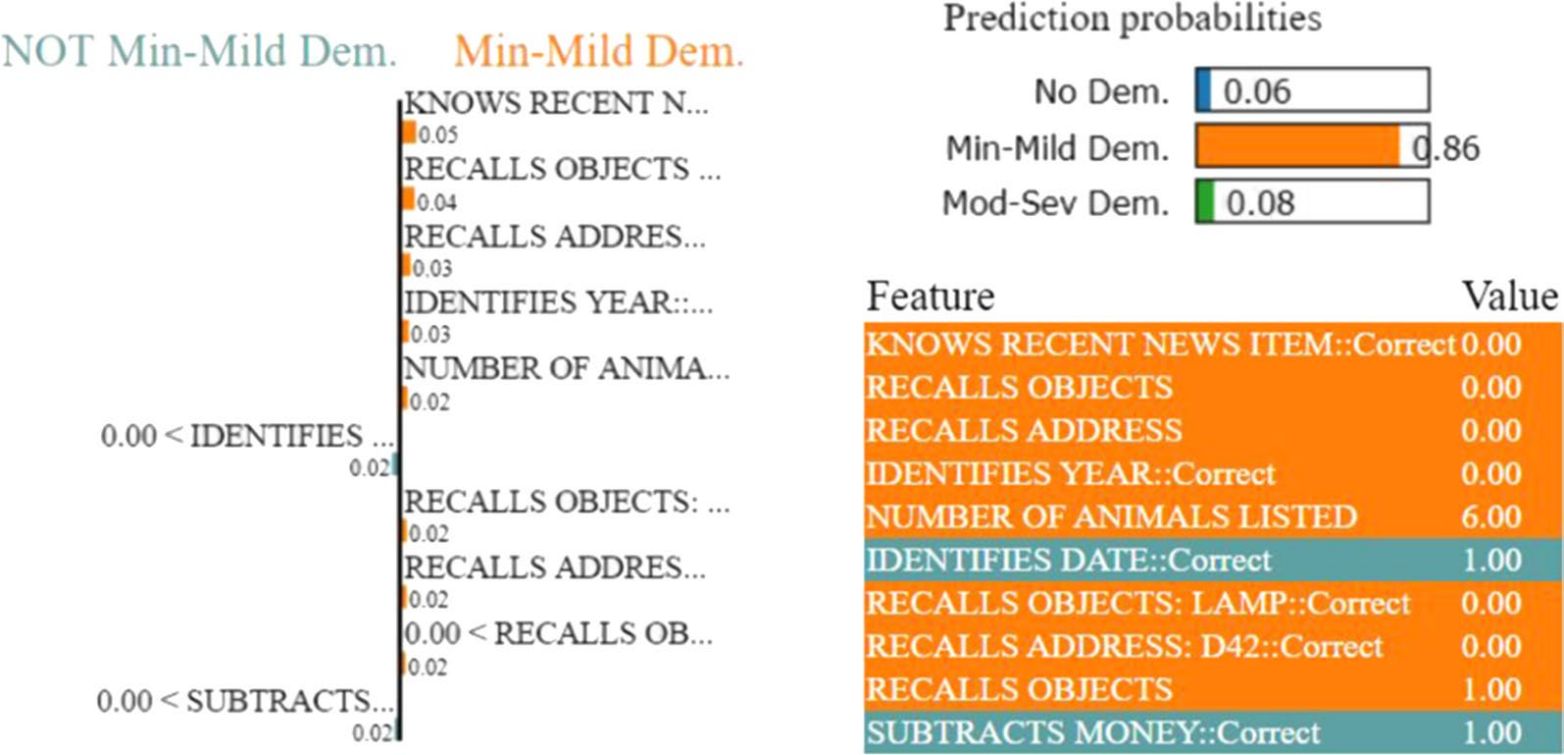
LIME provides the local interpretability for the prediction of an arbitrary test instance with prediction probabilities for Problem (b). The left part of the figure shows the weights of the top 10 important features while making class decision, whereas the right part represents predicted class probability and the top 10 important feature with their values. The weight of the feature represents its importance. Here, the short names of the impacting features in the left part of the figure are in the same order as in the feature & value list in the right part. Min-Mild Dem. and Mod-Sev Dem are the abbreviations for the Minimal or Mild dementia and Moderate or Severe dementia classes, respectively

**Fig. 10:**
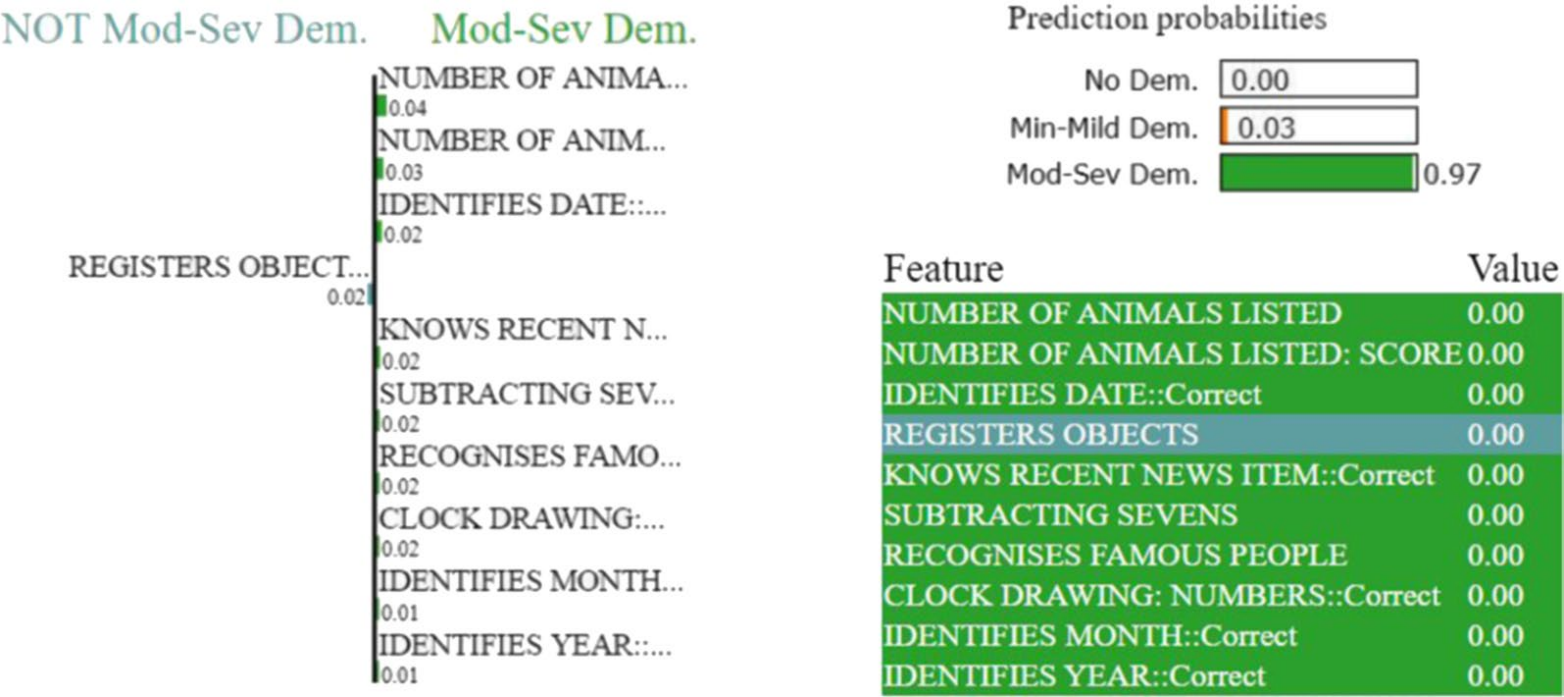
LIME provides the local interpretability for the prediction of an arbitrary test instance with prediction probabilities for Problem (b). The left part of the figure shows the weights of the top 10 important features while making class decision, whereas the right part represents predicted class probability and the top 10 important feature with their values. The weight of the feature represents its importance. Here, the short names of the impacting features in the left part of the figure are in the same order as in the feature & value list in the right part. Min-Mild Dem. and Mod-Sev Dem are the abbreviations for the Minimal or Mild dementia and Moderate or Severe dementia classes, respectively

Figures 7, 8, 9, and 10 depict the contribution of the top 10 features for predicting the class for the respective problems for a test instance. LIME calculates the contribution of each feature for the predicted class of the test instance in terms of weight. We only show top 10 features based on their weights in Figs. 7, 8, 9, and 10. The weight of the feature represents its importance. The left parts of the figures show the weights of the top 10 important features, while making class decisions, whereas the right parts of the figures represent the probabilities of the different classes, the names of the top 10 features and their values in the test instance. The weight’s importance can be interpreted by applying this to the prediction probabilities.

For example, in the right part of Fig. 10, features in green colours support the “Moderate or Severe” class and features in other colours support “No Dementia” and “Minimal or Mild dementia” classes. The left part of Fig. 10 measures the impact of these features in terms of weight while deciding for “NOT Moderate-Severe Dementia” (thus, “No-Dementia” or “Minimal or Mild Dementia”) and “Moderate-Severe Dementia”. If the features ‘NUMBER OF ANIMALS LISTED’ and ‘IDENTIFIES DATE’ are removed, the classifier should be able to predict class ’Moderate-Severe Dementia’ with a probability of $0.97-0.04-0.02 = 0.91$.

## Discussion

The biology and pathophysiology of dementia and its many underlying causes (e.g. Alzheimer’s disease that is the most common, at least in later life) are diverse and subject to the influence of different factors (e.g. comorbidity, lifestyle, and genetics). They demand individualised and precise treatment to differentiate the conditions of each dementia patient. The approach described in this paper has shown the unpredictability that derives from a complex interplay of factors impacts on the accuracy and efficiency of diagnosis. This complexity could be addressed using automated models based on machine learning, resulting in better performance of existing diagnostic tools.

Similar works also employ machine learning techniques aiming to address the most basic problem: predicting various dementias and/or detect probable dementia cases among undiagnosed patients analysing structured data features (prescribed medications, comorbidities etc.) from EMRs [16, 23] or even unstructured clinical notes [1]. Our study mainly differs in the variety, size, and granularity of the predictors utilized. In specific, we combine a huge set of demographic and clinical features with baseline arithmetic or memory tests, provided in the OPTIMA dataset. The most important of those predictors can be further used to produce a set of simple rules in the context of a decision tree, in order to assist a clinician in decision-making during the diagnostic process. These rules involve basic parameters (e.g. age) and simple cognitive tests (e.g. ‘Identifies date’) that can be easily applied by a medical expert to receive a prediction for an individual patient with a certain confidence. Visual models as such can assist in making our results interpretable by the experts, in contrast to decisions made by black-box machine learning tools. Moreover, our study applies the same approach to address a more advanced multi-class problem: detecting the presence and the severity level of dementia in the same patient cohort. This problem is also effectively addressed using a random forest model, and a similar set of simple rules is provided via a second decision tree.

Since the OPTIMA dataset is based on the London population, location and time have a substantial impact on numerous features like “Remembers Lindbergh”, “Recognise Picture Barometer”, “Remembers WW II Date”, and “Knows Prime Minister”. These features should alter in name depending on the location and time, but the essence of each feature will remain the same. In the United States, for example, “Knows Prime Minister” feature should be translated to “Knows President” (i.e. recognizing the most powerful political position). As a result, our models aid the recognition of features based on inherent nature, but they must be retrained for new locations and periods.

Improvements in the diagnostic process will significantly enhance a clinician’s ability to offer the management plan most appropriate to an individual patient, both at present and when disease-modifying treatments become available for sporadic neurodegenerative dementia. To be given a diagnosis of any untreatable condition is a life-changing event. When the condition is neurodegenerative dementia, patients need to make changes to their current lives and future plans, to consider legal rights appropriate to their future selves, and if possible, maximise the utility of their residual cognitive resources. While the disease remains untreatable, there are negative as well as positive aspects to early diagnosis, and where accuracy is concerned, it is more important to avoid false-positive than false-negative diagnoses. However, the advent of disease-modifying treatments will change this: identifying a neurodegenerative condition as early as possible in the course of its evolution will limit the damage already done and therefore improve the outcome of treatment. At the same time, it will become as important not to miss a true positive as to misclassify a true negative.

Once diagnosed, a patient with Alzheimer’s disease needs to be kept under regular clinical assessment. Whether conducted in the context of primary care or a specialist clinic, ongoing assessment aims to ensure that the patient’s social and medical needs (including the choice and dose of symptomatic treatments) are optimally aligned with their cognitive abilities. Because current methods of assessment are both time-consuming and prone to inaccuracy and error, an a priori estimate of the likely trajectory of decline would alert the clinician to any anomalous results, and therefore, ensure that the best decisions are always made at the most appropriate time.

## Conclusions and future directions

This paper presented automated prediction models for detecting the presence of dementia in the Electronic Medical Records of patients of a large ageing study, based on psychometric tests and demographic factors. Our study focused both on the accuracy, by employing different machine learning techniques, and interpretability, by visualising resulting models with the method of decision trees. The decision trees produced identified the most discriminating—and thus important—features for dementia detection, as well as for the disease severity classification. Cognitive test features seem to be the most relevant, including various memory (e.g. recollection of objects) and arithmetic (e.g. subtraction of money) tasks that patients have been asked to take, with their performance determining the sub-group in which they fall with certain probability. Sub-groups define either a specific severity level of the disease or a non-dementia condition (e.g. patients with MCI). The predictive models assist the clinician in determining the order in which the most relevant questions should be addressed while assessing a patient’s cognitive abilities.

Our work aims at thoroughly investigating and highlighting key characteristics that yield the presence and severity of dementia and creating an accurate prediction tool. Moreover, the decision tree approach ignores mainstream cognitive tests such as MMSE and CAMCOG, employed in most of the related work, in order to focus on simple rules, represented by simple arithmetic and memory tasks. Such a rule-based tool can easily assist clinicians in the early detection of dementia in primary care. To adopt this approach, the end-user of this tool would only have to assign the tasks depicted in the decision trees to her patients, in order to assess their condition with a certain confidence (based on the proportion of each sub-group in the tree). Thus, we hope that these results and tool can represent building blocks for individualised clinical decisions (Additional file 1 and 2: Figures S1 and S2).

As for future challenges, we plan to validate the proposed prediction models, including random forests and interpretable decision trees, in other patient cohorts. An extensive evaluation across different populations would ensure the current approach does not suffer from a lack of scientific validity. More importantly, an extensive assessment will provide empirical proof of the generality of the properties of the proposed methods.

## Supplementary information

**Additional file 1: Figure S1.**The decision tree predicts the dementia classes ("Dementia"/"No Dementia") of 2505 patients for the Problem (a). This figure file provides high resolution for better visibility.

**Additional file 2: Figure S2.**The decision tree predicts the dementia classes ("No Dementia"/"Minimal or Mild Dementia"/"Moderate or Severe Dementia") of 2505 patients for the Problem (b). This figure file provides high resolution for better visibility.

**Additional file 3: Figure S3.**Decision Tree predicts the dementia classes ("Dementia"/"No Dementia") for the Problem (a) using baseline method. The decision trees differ in appearance as well as decision thresholds for certain non-interpretable attributes

**Additional file 4: Figure S4.**Decision Tree predicts the dementia classes ("No Dementia"/"Minimal or Mild Dementia"/"Moderate or Severe Dementia") for the Problem (b) using baseline method. The decision trees differ in appearance as well as decision thresholds for certain non-interpretable attributes

**Additional file 5: Table S1.**Optima dataset feature guide in excel format

## Data Availability

Code is available on www.github.com/SDM-TIB/dementia_detection.git. The data that support the findings of this study were provided from the Oxford Project to Investigate Memory and Ageing (OPTIMA) via a bilateral agreement with the IASIS project and cannot be publicly shared.

## Abbreviations

BMI: Body mass index
CAMCOG: Cambridge Cognition Examination
CAMDEX: Cambridge Mental Disorders of the Elderly Examination
CDT: Clock Drawing Test
DT: Decision Tree
EMR: Electronic Medical Record
ICD: International Classification of Diseases
LIME: Local Interpretable Model-agnostic
MCI: Mild Cognitive Impairment
OPTIMA: Oxford Project to Investigate Memory and Ageing
MMSE: Mini-Mental State Examination

## Footnotes

1 https://scikit-learn.org/stable/.
