# Supplementary material for: Identifying the presence and severity of dementia by applying interpretable machine learning techniques on structured clinical records
Source: BMC Med Inform Decis Mak. 2022 Oct 17;22:271. doi: 10.1186/s12911-022-02004-3 (PMC9578246; doi:10.1186/s12911-022-02004-3)

Figure S1: The decision tree predicts the dementia classes (``Dementia''/``No Dementia'') of 2,505 patients for the Problem (a). The pie charts in leaves show class labels, the proportion of resulting classes and their support size. The branches demonstrate connections between features and their threshold values, leading towards class labels.

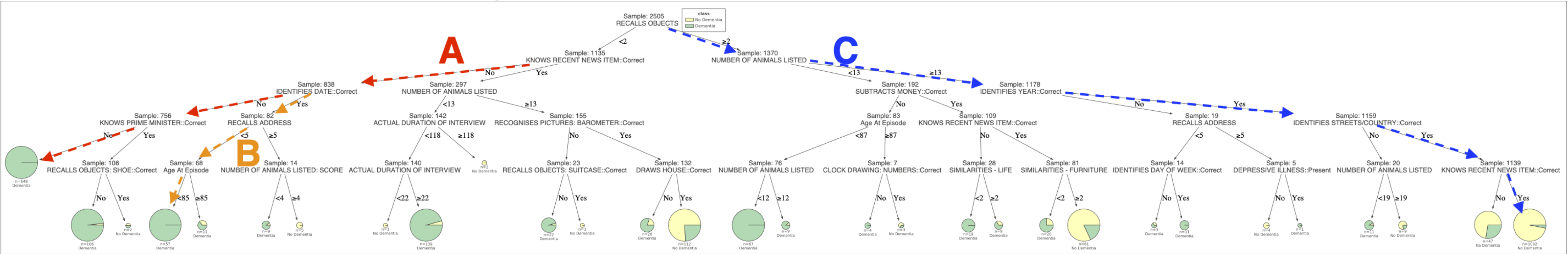

Supplement: Supplementary file 1 — Additional file 1: Figure S1.The decision tree predicts the dementia classes ("Dementia"/"No Dementia") of 2505 patients for the Problem (a). This figure file provides high resolution for better visibility. [file 12911_2022_2004_MOESM1_ESM.pdf]
