# Supplementary material for: Identifying the presence and severity of dementia by applying interpretable machine learning techniques on structured clinical records
Source: BMC Med Inform Decis Mak. 2022 Oct 17;22:271. doi: 10.1186/s12911-022-02004-3 (PMC9578246; doi:10.1186/s12911-022-02004-3)

Figure S2: The decision tree predicts the dementia classes (``No Dementia''/ ``Minimal or Mild Dementia'' / ``Moderate or Severe Dementia'') of 2,505 patients for the Problem (b). The pie charts in leaves show class labels, the proportion of resulting classes and their support size. The branches demonstrate connections between features and their threshold values, leading towards class labels

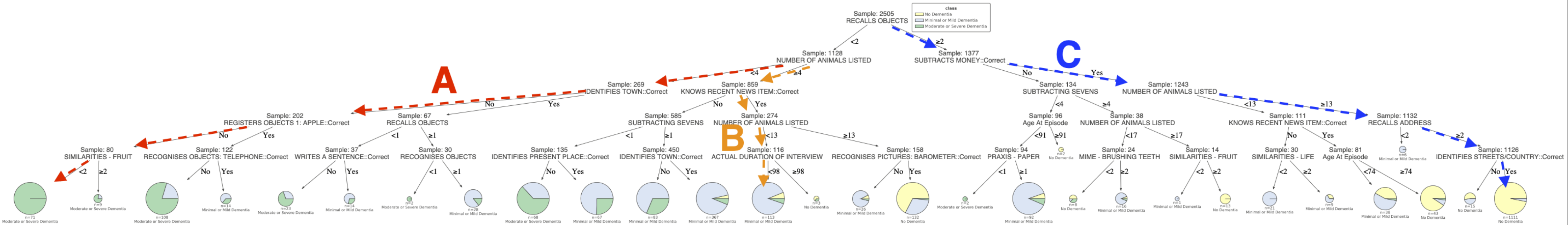

Supplement: Supplementary file 2 — Additional file 2: Figure S2.The decision tree predicts the dementia classes ("No Dementia"/"Minimal or Mild Dementia"/"Moderate or Severe Dementia") of 2505 patients for the Problem (b). This figure file provides high resolution for better visibility. [file 12911_2022_2004_MOESM2_ESM.pdf]
