# Supplementary material for: Identifying the presence and severity of dementia by applying interpretable machine learning techniques on structured clinical records
Source: BMC Med Inform Decis Mak. 2022 Oct 17;22:271. doi: 10.1186/s12911-022-02004-3 (PMC9578246; doi:10.1186/s12911-022-02004-3)

Figure S3: Decision Tree predicts the dementia classes (``Dementia''/``No Dementia'') for the Problem (a) using baseline method. The decision trees differ in appearance as well as decision thresholds for certain non-interpretable attributes. The pie charts in leaves show class labels, the proportion of resulting classes and their support size. The branches demonstrate connections between features and their threshold values, leading towards class labels

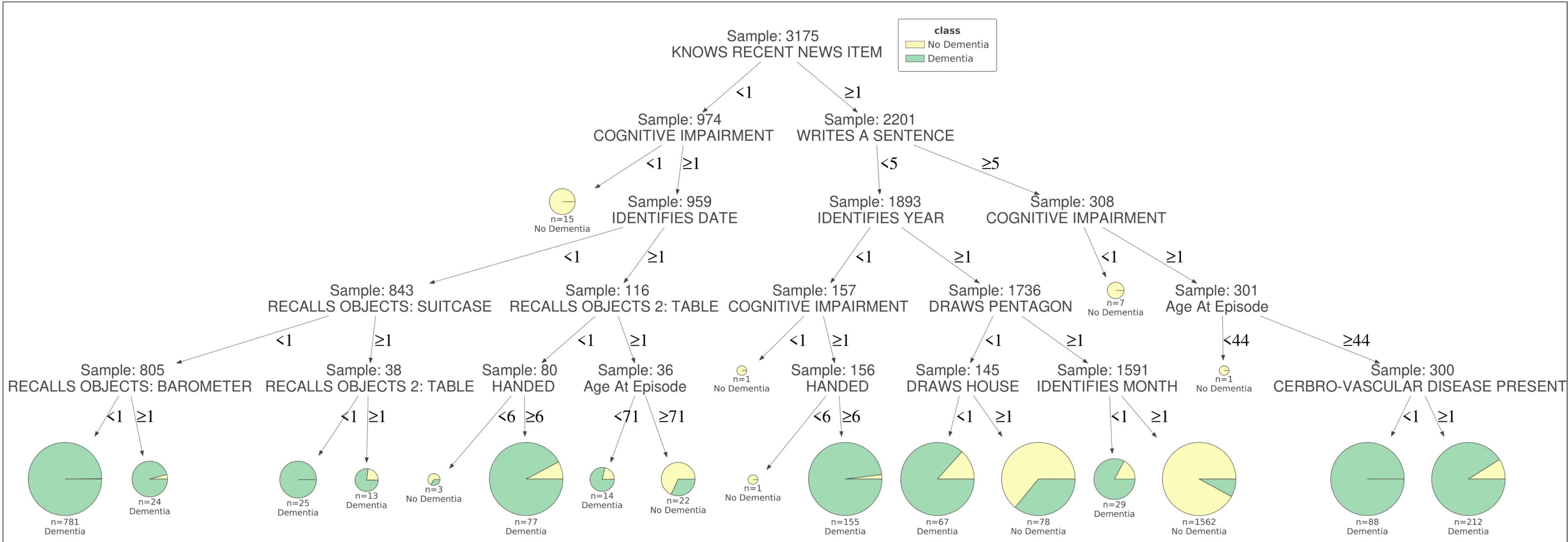

Supplement: Supplementary file 3 — Additional file 3: Figure S3.Decision Tree predicts the dementia classes ("Dementia"/"No Dementia") for the Problem (a) using baseline method. The decision trees differ in appearance as well as decision thresholds for certain non-interpretable attributes [file 12911_2022_2004_MOESM3_ESM.pdf]
